# In Crohn's Disease, Anti-TNF-**α** Treatment Changes the Balance between Mucosal IL-17, FOXP3, and CD4 Cells

**DOI:** 10.5402/2012/505432

**Published:** 2012-06-14

**Authors:** Veera Hölttä, Taina Sipponen, Mia Westerholm-Ormio, Harri M. Salo, Kaija-Leena Kolho, Martti Färkkilä, Erkki Savilahti, Outi Vaarala, Paula Klemetti

**Affiliations:** ^1^Immune Response Unit, Department of Vaccination and Immune Protection, National Institute for Health and Welfare, Haartmaninkatu 8, 00290 Helsinki, Finland; ^2^Division of Gastroenterology, Department of Medicine, Helsinki University Hospital, Haartmaninkatu 4, 00290 Helsinki, Finland; ^3^Children's Hospital, University of Helsinki, Stenbäckinkatu 11, 00290 Helsinki, Finland

## Abstract

*Aim*. In Crohn's disease (CD), anti-TNF-*α* treatment is a potent medication. We aimed to characterize the effect of anti-TNF-*α* treatment on T effector and regulatory cells. *Material and Methods*. We studied T-effector and regulatory cells on cellular and mRNA levels in intestinal biopsy samples from 13 Crohn's disease patient. Biopsies were obtained at baseline and 3 months after anti-TNF-*α* treatment, and from 14 inflammation-free control subjects. *Results*. Patients had higher numbers of ileal IL-17^+^ and forkhead box P3 (FOXP3)^+^ cells than did control subjects, both before ( *P* ≤ 0.001 and *P* ≤ 0.05, resp.) and after the anti-TNF-*α* treatment (*P* ≤ 0.01, *P* ≤ 0.01). Intestinal interferon-*γ* and IL-17 mRNA expression was higher in Crohn's disease and remained elevated after anti-TNF-*α* treatment. The ratio of IL-17^+^ cells to CD4^+^ cells decreased (*P* ≤ 0.05) and compared to baseline the ratio of IL-17^+^ cells to FOXP3^+^ was lower after treatment (*P* ≤ 0.05). *Conclusions*. TNF-*α*-blocking agents improved intestinal balance between IL-17^+^ T-effector and regulatory T cells, although intestinal IL-17 upregulation remained elevated.

## 1. Introduction

Chronic intestinal inflammation characterizes Crohn's disease (CD), the etiology of which is incompletely understood, but likely intestinal microflora components cause aberrant immune responses. Discovery of *NOD2* genetic polymorphism has identified *CARD15* allele as a risk factor for CD and supports the important role of the innate immune system in CD pathogenesis. Interleukin 23 receptor (*IL-23R*) gene polymorphisms associate with CD and provides a link to Th17 immunity, a hallmark of CD [1–4]. IL-23 is a cytokine important for the expansion and function of IL-23R-expressing Th17 cells [5]. In CD, colonic and ileal biopsy samples show IL-17^+^ cells [3, 4, 6]. 

Biological agents contribute to the repertoire of medical treatment of CD: tumor necrosis factor- (TNF-) *α* blocking therapy is effective in inducting and maintaining remission [7, 8]. In active CD, infliximab induces rapid clinical improvement as well as rapid endoscopic and histological healing [9]; unfortunately, the cessation of anti-TNF-*α* therapy tends to lead to a disease relapse. Conventional therapies fail to normalize the numbers of IL-17^+^ cells in the ileal lamina propria (LP), even during endoscopic remission [4]. Colitogenic Th17 cells may be a factor behind the relapsing nature of the disease [10]. Whether TNF-*α*-blocking therapy normalizes the number of intestinal Th17 cells remains unknown. Few studies of infliximab's effect on Th17 cells exist in rheumatoid arthritis infliximab, which seems to reduce peripheral Th17 cells [11]. 

Transcription factor FOXP3 characterizes regulatory T cells (Tregs), a unique subset of T cells that regulate immune response [12–14]. A few studies on Tregs and their role in intestinal inflammation exist and most of them show a consistent expansion of intestinal Tregs and suppressive Treg activity *in vitro* [15–17]. In adult CD, however, the effect of TNF-*α*-blocking therapy on intestinal Tregs is unknown. Further, in IBD, the balance between Th17 and Treg cells plays an important role [10].

## 2. Materials and Methods

### 2.1. Patients

In Helsinki University Central Hospital 13 adult patients with established CD twice underwent ileocolonoscopy: a baseline endoscopy and a treatment-response evaluation at 12 weeks. The Crohn's disease endoscopic index of severity (CDEIS) served to grade the endoscopy findings: scores <3 suggested inactive disease and ≥3 endoscopically active disease [18, 19]. The Crohn's disease activity index (CDAI) served to assess clinical activity [14]. At the time of endoscopies, patients provided blood and fecal samples. After the baseline endoscopy, all patients received anti-TNF-*α*-therapy (median 7 days after, range 1–38 days). 12 received infliximab infusions 5 mg/kg at weeks 0 and 8, and one patient received an adalimumab induction dose 80 mg subcutaneously at week 0, followed by 40 mg subcutaneously every other week until week 8; thereafter she underwent the second endoscopy at 10 weeks. Before the anti-TNF-*α* therapy, maintenance therapy remained unchanged, thereafter corticosteroids were tapered off.  Table 1 shows patient characteristics.

The control group comprised 14 patients referred to ileocolonoscopy for the following indications: change in bowel habits in 3, diverticular disease, colorectal cancer followup, history of adenoma, and rectal bleeding (2 with each), history of polyps in one, exclusion of CD in one patient with history of perianal abscesses, and in one patient with abdominal pain. In routine histological analysis of biopsies, all control subjects showed normal findings.

### 2.2. Immunoenzymatic Labeling and Microscopic Evaluation

During endoscopies, two mucosal biopsies were taken, frozen in OCT, and stored at −70°C. For immunohistochemical staining, 7 *μ*m sections were cut, with the avidin-biotin immunoperoxidase system applied according to the Vectastain ABC Elite kit (Vector Laboratories, Peterborough, UK) instructions. The acetone-fixed slides were blocked in normal serum for 30 minutes. We incubated slides for 1 hour with either monoclonal mouse anti-human CD4 (BD Pharmingen, San Diego, CA, USA), CD8 (Dakocytomation, Glostrup Denmark), FOXP3 (Abcam, Cambridge, UK), or polyclonal rabbit anti-human IL-17 (Santa Cruz Biotechnology, Santa Cruz, CA, USA) antibodies. Then the slides were incubated in biotinylated antibody and ABC reagent. AEC served as a chromogen and hematoxylin served to counterstain the slides. Omission of the primary antibodies served as the negative control. The numbers of positively stained LP cells were counted under a Leica DM4000B light microscope through a calibrated graticule. In each specimen, positive cells were expressed as the mean number of cells/mm^2^.

### 2.3. Fecal Calprotectin Assays

The values quoted as normal fecal calprotectin were <100 *μ*g/g of stool [15, 16] and were measured by a quantitative enzyme immunoassay (PhiCal Test, Calpro AS, Oslo, Norway).

### 2.4. Quantitative Real-Time PCR

Total RNA was extracted from biopsies with the GenElute Mammalian total RNA miniprep kit (Sigma, St. Louis, MO, USA) and reverse transcribed as earlier described [4]. QPCR runs with cDNA samples and TaqMan reagents (Applied Biosystems) were performed in triplicate on the StepOnePlus RT-PCR system (Applied Biosystems)programmed for an initial step of 20 seconds at 95°C, followed by 40 thermal cycles of 1 second at 95°C and 20 seconds at 60°C. Raw data were analyzed as previously described [4]. Target-specific primers/probes were IL-17A, FOXP3, IL-23A p19, TNF-*α*, and 18s (Applied Biosystems). Ribosomal 18s RNA served as the endogenous control.

### 2.5. Statistical Analysis

For all studies, data are expressed as median (range). The nonparametric Kruskal-Wallis test, the Mann-Whitney *U*-test, Wilcoxon signed rank test, and Spearman's correlation served for statistical analyses (SPSS, v14.0; SPSS Inc, Chicago, IL, USA). A *P* value < 0.05 was considered significant. 

### 2.6. Ethical Considerations

The Ethics Committee, Department of Medicine, Helsinki University Central Hospital, Helsinki, approved the study plan. All patients and control subjects gave their informed written consent to participate in this study.

## 3. Results

### 3.1. IL-17^+^, FOXP3^+^, CD4^+^, and CD8^+^ Cells in Biopsy Samples

**Figure 1:**
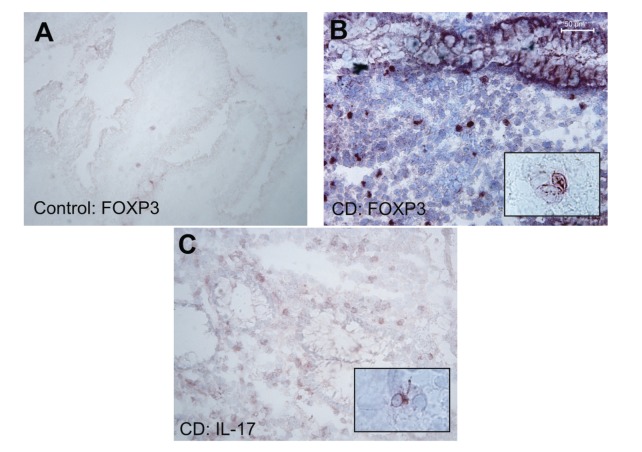
Immunoperoxidase staining for forkhead box P3 (FOXP3) and interleukin 17 (IL-17) in frozen sections, 3-amino-9-ethylcarbazole-hematoxylin stain. Staining of FOXP3 in mucosal biopsy specimens from a control patient (a), original magnification 200x. Representative stainings for FOXP3 (b, nuclear) and IL-17 (c, paranuclear) from a Crohn's disease patient. Scale bar indicates 50 *μ*m, original magnifications 100x and 200x. Insets: FOXP3 (b) and IL-17 (c), original magnification 1000x.

**Figure 2:**
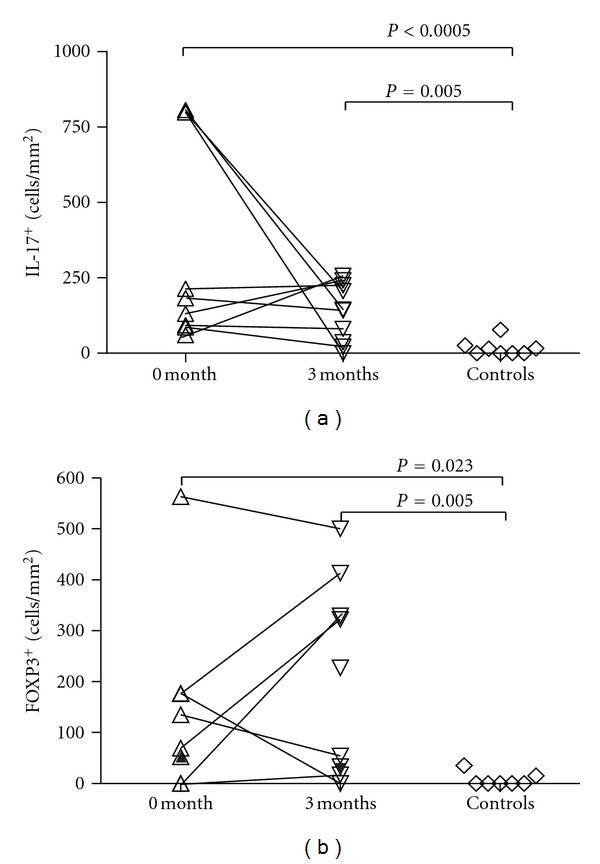
Changes in numbers of IL-17^+^ and FOXP3^+^ cells in ileal biopsy samples taken before 0 and 3 months after beginning of anti-TNF-*α* treatment (a, b).

Figure 1 shows IL-17 and FOXP3 stainings. For statistical analyses, patients with disease localization differing from the sample site were excluded. The number of ileal IL-17^+^ cells was higher in CD than in the control subjects before and after treatment (*P* < 0.0005 and 0.005, Mann-Whitney *U*-test, Figure 2(a)), and the number of ileal FOXP3^+^ cells was higher in patients' samples both before and after treatment (*P* = 0.023 and 0.005, Figure 2(b)). The number of colonic IL-17^+^ cells was higher in CD at baseline and 3-month follow-up samples than in controls (*P* = 0.015 and 0.042). The number of colonic FOXP3^+^ cells was higher in CD in the 3-month samples than in controls (*P* = 0.032). Numbers of ileal or colonic CD4^+^ or CD8^+^ cells showed no difference. In the parameters measured no difference emerged between baseline and 3-month follow-up samples.

### 3.2. mRNA Expression in Biopsy Samples (Table 2)

Colonic IL-17 mRNA expression was higher in CD than in controls at baseline (*P* = 0.038), but not after treatment. Ileal IL-23 mRNA expression was higher both before and after treatment (*P* = 0.037 and 0.022). In CD, ileal and colonic IFN-*γ*, along with ileal FOXP3 mRNA expression showed an increase before and after treatment. 

### 3.3. Relative Number of IL-17^+^ Cells or FOXP3^+^ Cells of CD4^+^ and CD8^+^ Cells

**Figure 3:**
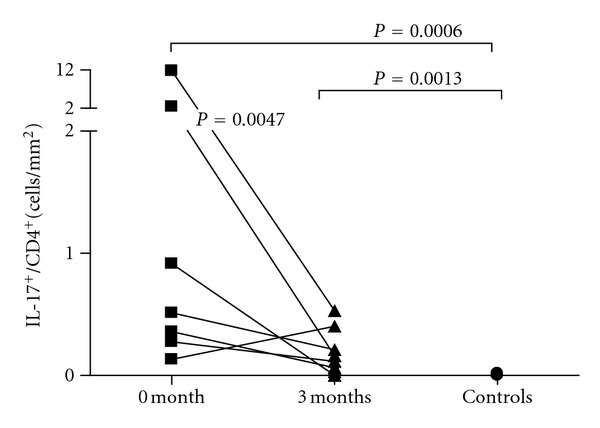
Ratio of IL-17^+^ cells to CD4^+^cells in ileal biopsy samples in active CD before 0 and 3 months after beginning of anti-TNF-*α* treatment and control subjects.

Compared to the controls, the patients showed an increased ratio of ileal IL-17^+^ cells to CD4^+^ cells in both samples (*P* = 0.0006 and 0.0013, Figure 3). Anti-TNF-*α* treatment reduced significantly the ratio of IL-17^+^ cells to CD4^+^ cells (*P* = 0.047, Wilcoxon signed rank test). In both samples, the ratio of ileal IL-17^+^ cells to CD8^+^ cells was higher in CD than in controls (*P* = 0.0001 and *P* = 0.0031). The ratio of ileal FOXP3^+^ cells to CD4^+^ cells was higher in both samples in CD than in controls (*P* = 0.03 and 0.03). In the parameters measured no difference appeared between the two samples.

### 3.4. Relation between T Effector and T Regulatory Cell Markers

**Figure 4:**
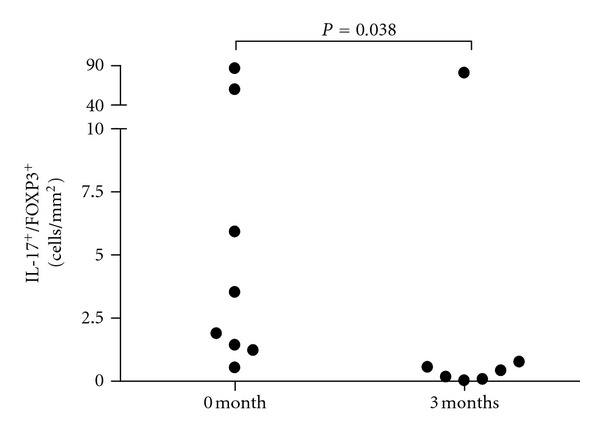
Ratio of IL-17^+^ cells to FOXP3^+^ cells higher in 0-month samples than in 3-month samples (*P* = 0.038, Mann-Whitney *U*-test).

The ratio of ileal IL-17^+^ cells to FOXP3^+^ cells was higher at baseline than in 3-month samples (*P* = 0.038, Mann-Whitney *U*-test, Figure 4). The ratio of ileal or colonic IL-17 or IFN-*γ* mRNA expression to FOXP3 mRNA expression showed no difference between the two samples. In CD, ileal and colonic IFN-*γ* and IL-17 mRNA expression correlated positively (*r* = 0.636; *P* = 0.026; *r* = 0.539; *P* = 0.021).

### 3.5. Correlation between CDEIS and Markers for T Effector or T Regulatory Cells

At treatment-response endoscopy ileal IL-17^+^ cells and IL-17 mRNA expression correlated positively with CDEIS (*r* = 0.806, *P* = 0.007; *r* = 0.736, *P* = 0.038). FOXP3^+^ or CD4^+^ cells and CDEIS showed no correlation, but at baseline ileal FOXP3 mRNA expression and CDEIS correlated positively (*r* = 0.830; *P* = 0.041). 

## 4. Discussion 

In CD, a marked decrease in CDEIS associated with a decreasing ratio of intestinal IL-17^+^ cells related to the numbers of CD4^+^ cells and FOXP3^+^ cells. These results suggest that anti-TNF-*α* treatment leads to an improved balance between regulatory T cells and IL-17 effector T cells. The relative upregulation of regulatory mechanisms is likely the explanation for the clinical and endoscopic response to treatment since the TNF-*α* blockade failed to normalize the mucosal IL-17 or IFN-*γ* upregulation that is characteristic of CD [3, 4]. These findings support the view of the synergistic role of IL-17 and TNF-*α* in tissue damage. 3 months after treatment, CDEIS correlated positively with magnitude of intestinal IL-17 immunity, indicating that when the effect of TNF-*α* is blocked, the role of IL-17 in the intestinal inflammation is clearly demonstrable. Before the anti-TNF-*α* treatment, no such correlation existed between CDEIS and IL-17 immunity. Accordingly, full endoscopic remission associated with lower IL-17 activity after treatment, whereas the only patient with increasing CDEIS during treatment showed an increase also in her proportion of IL-17^+^ cells. On the other hand, although anti-TNF-*α* treatment was unable to downregulate directly the IL-23/IL-17 axis responsible for mucosal damage, its beneficial effect was mediated via changes in the immunological network. 

 Full understanding of the relation between Th17 and T regulatory cells is lacking. TNF-*α* suppresses the development of Th17 cells from naïve CD4 cells [20], but the function of T regulatory cells complicates the interaction; blocking the function of TNF-*α* does not downregulate IL-17 immunity. This correlates with our findings suggesting a relative decrease in IL-17 immunity in relation to total number of CD4^+^ cells and FOXP3^+^ cells. In addition, IL-17^+^ cells may arise from Tregs due to their plasticity. In the presence of IL-6, CD4^+^CD25^+^FOXP3^+^ cells can differentiate into Th17 [21], however, anti-TNF-*α* treatment reduces IL-6 production and may inhibit this differentiation. The beneficial change in the balance between IL-17^+^ and FOXP3^+^ cells may reflect this phenomenon. 

Because CD4^+^CD25^−^ cells can be induced to express FOXP3 [22], intestinal FOXP3 expressing cells may be either classical Treg or activated T cells. Activated FOXP3-expressing T cells are hyporesponsive, with impaired suppressive function, and transient FOXP3 expression also exists in a nonsuppressive T cell population [23]. Possibly in active CD, T cell activation induces FOXP3 expression and thus does not necessarily display suppressive capability. In remission, a high number of FOXP3^+^ cells may, however, reflect the number of natural, suppressive T regulatory cells.

In evaluating the effect of anti-TNF-*α* agents on immunological markers, changes in maintenance therapy are important. Most of our patients received corticosteroid treatment at the first administration of anti-TNF-*α* treatment, but none at the time of the second colonoscopy. Corticosteroids inhibit T cell activation by multiple mechanisms [24], possibly through induction of T regulatory cells. In asthma patients receiving glucocorticoid treatment, FOXP3 mRNA expression increases [25]. In keeping with this, we report increased numbers of FOXP3^+^ cells in active disease, but here FOXP3 was high also after treatment.

Especially in animal models for inflammatory bowel disease, evidence accumulates regarding the importance of the IL-23/IL-17 axis [26]. A unique subset of intestinal macrophages producing proinflammatory cytokines such as IL-23, TNF-*α*, and IL-6 exists [27]. In CD, the number of these macrophages is higher and they contribute to IFN-*γ* production by LP mononuclear cells rather than IL-17, and IFN-*γ* triggers further abnormal macrophage differentiation with hyperproduction of IL-23. Likewise, we showed enhanced ileal IFN-*γ* mRNA expression in CD. Th1/Th17 mucosal cells and T cell lines able to secrete both IL-17 and IFN-*γ* have been described in CD related intestinal inflammation [28, 29]. A positive correlation between IL-17 and IFN-*γ* mRNA expression in ileal CD may reflect activation of these kinds of cells. Possibly, Th1/Th17 cells produced IL-17 and IFN-*γ*, but because we lacked material to further characterize IL-17- or IFN-*γ*-producing cells, this question remains open. Unfortunately, we were unable to accomplish double-staining for IL-17 or FOXP3, and for surface markers, due to lack of material.

Our data showed that mucosal healing induced by TNF-*α* blocking treatment associates with beneficial changes in the balance between regulatory and effector T cells. The presence of Th17 cells after the treatment suggests the need for maintenance therapy to avoid the risk of relapse. 

## Figures and Tables

**Table 1:** Patient characteristics.

	*n*
Gender: female/male	6/7 (46/54%)
Disease type	
Inflammatory	6 (46%)
Stricturing	4 (31%)
Inflammatory + perianal	3 (23%)
Disease location	
Ileum	2 (15%)
Colon	3 (23%)
Ileocolon	8 (62%)
Medication (at first endoscopy)	
Azathioprine or 6-	9 (69%)
mercaptopurin	2 (15%)
Methotrexate	9 (69%)
Corticosteroids	9 (69%)
Mesalamine	2 (15%)
Salazosulphapyridine	1 (8%)
Metronidazole	
Medication (at second endoscopy)	
Azathioprine or 6-	11 (85%)
mercaptopurin	2 (15%)
Methotrexate	0 (0%)
Corticosteroids	8 (62%)
Mesalamine	2 (15%)
Salazosulphapyridine	0 (0%)
Metronidazole	
Previous anti-TNF-*α*	4 (31%)
treatment	

	Median (range)

Age, years,	23 (19–44)
Disease duration, years	5.1 (0.4–27.0)
CDEIS	
Before treatment	14.4 (1.8–25.3)
After treatment	4.4 (0.0–11.2), *P* = 0.006
CDAI	
Before treatment	174 (50–605)
After treatment	64 (24–112), *P* = 0.003
Fecal calprotectin *μ*g/g	
Before treatment	1173 (88–15326)
After treatment	127 (13–1419), *P* = 0.002
Serum CRP	
Before treatment (*n* = 10)	8 (<5–42)
After treatment	<5 (<5-6), *P* = 0.012

CDAI: Crohn's disease activity index; CDEIS: Crohn's disease index of severity.

**Table 2:** mRNA expression of L-17, IL-23, FOXP3, IFN-*γ* and TNF-*α* in ileal and colonic biopsy samples taken before (0) and 3 months after the beginning of anti-TNF-*α* treatment. Median (range) mRNA levels are expressed as relative units in relation to in-house calibrator (phytohemagglutinin-stimulated peripheral blood monocytes). Asterix: statistically significant differences between patients and control subjects.

		0 month	3 months	Controls

Ileum	IL-17	128.0 (30–325)	67.5 (30–615)	30.0 (30–419)
	IL-23	373.0 (322–804)^∗^	444.5 (235–542)^∗^	225.0 (153–706)
	FOXP3	611.0 (15–792)^∗^	104.5 (15–891)^∗^	20.5 (15–220)
	IFN-*γ*	19.6 (8.6–230)^∗∗∗^	14.0 (1.00–37.6)^∗^	1.0 (1.0–6.0)
	TNF-*α*	38.7 (8.6–206.6)	43.7 (10.2–249.9)	—

Colon	IL-17	133.5 (30–588)^∗^	79.0 (30–1607)	30.0 (30–144)
	IL-23	167.0 (72–405)	240.0 (74–983)	252.0 (9–392)
	FOXP3	311.5 (63–694)^∗∗^	135.5 (15–1284)	49.0 (15–252)
	IFN-*γ*	45.0 (2.0–249.7)^∗∗^	15.7 (1.0–122.2)^∗^	1.0 (1.0–5.6)
	TNF-*α*	30.9 (8.9–53.4)	23.9 (3.1–111.7)	18.8 (3.4–47.6)

*P < 0.05, **P < 0.01, ***P < 0.001, Mann-Whitney *U*-test.

## Abbreviations

CD: Crohn's disease
CDAI: Crohn's disease activity index
CDEIS: Crohn's disease index of severity
IL: Interleukin
IFN: Interferon
LP: Lamina propria
TNF: Tumour necrosis factor.
